# Depression, Anxiety, and Stress Among Healthcare Workers During the COVID-19 Outbreak and Relationships With Expressive Flexibility and Context Sensitivity

**DOI:** 10.3389/fpsyg.2021.623033

**Published:** 2021-02-22

**Authors:** Vittorio Lenzo, Maria C. Quattropani, Alberto Sardella, Gabriella Martino, George A. Bonanno

**Affiliations:** ^1^Department of Social and Educational Sciences of the Mediterranean Area, University for Foreigners “Dante Alighieri” of Reggio Calabria, Reggio Calabria, Italy; ^2^Sisifo – Consortium of Social Cooperatives, Catania, Italy; ^3^Department of Clinical and Experimental Medicine, University of Messina, Messina, Italy; ^4^Teachers College, Columbia University, New York, NY, United States

**Keywords:** COVID-19, clinical psychology, depression, anxiety, stress, emotion regulation, flexibility, context sensitivity

## Abstract

This study aimed at investigating depression, anxiety, and stress symptoms among healthcare workers and examine the role of expressive flexibility and context sensitivity as key components of resilience in understanding reported symptoms. We hypothesized a significant and different contribution of resilience components in explaining depression, anxiety, and stress. A total sample of 218 Italian healthcare workers participated in this study through an online survey during the lockdown, consequently to the COVID-19. The Depression Anxiety Stress Scales-21 (DASS-21) was used to measure depression, anxiety, and stress; the Flexible Regulation of Emotional Expression (FREE) scale was used to measure the ability to enhance and suppress emotional expression; the Context Sensitivity Index (CSI) was used to measure the ability to accurately perceive contextual cues and determine cue absence. Demographic and work-related data were also collected. DASS-21 cut-off scores were used to verify the mental status among the respondents. Correlational analyses examined relationships between DASS-21, FREE, and CSI, followed by three regression analyses with depression, anxiety, and stress as dependent variables, controlling for age, gender, and work experience. Enhancement and suppression abilities, cue presence, and cue absence served as independent variables. The results showed a prevalence of moderate to extremely severe symptoms of 8% for depression, 9.8% for anxiety, and 8.9% for stress. Results of correlational analysis highlighted that enhance ability was inversely associated with depression and stress. Suppression ability was inversely associated with depression, anxiety, and stress. The ability to perceive contextual cues was inversely associated with depression and anxiety. The regression analysis showed that the ability to enhance emotional expression was statistically significant to explain depression among healthcare workers. In predicting anxiety, age, and the ability to accurately perceive contextual cues and determine cue absence made substantial contributions as predictors. In the last regression model, age, work experience, and the ability to suppress emotional expression were significant predictors of stress. This study’s findings can help understand the specific contributions of enhancement and suppression abilities and sensitivity to stressor context cues in predicting depression, anxiety, and stress among healthcare workers. Psychological interventions to prevent burnout should consider these relationships.

## Introduction

Since the end of February 2020, the number of confirmed cases of coronavirus disease 2019 (COVID-19) has dramatically ascended in Italy causing 78.755 deaths as of 10th January 2021 (Italian Ministry of Health, 2021). On March 9, the Italian Government adopted a massive lockdown to decrease the spread of the virus. Early studies have documented the psychological impact of this unprecedented decision concerning the Italian population. A study involving a sample of 6,314 Italian people showed that about a third of participants reported moderate to extremely severe depression, anxiety, and stress (Lenzo et al., 2020b). Another study found that more than half of the Italian population suffered impaired sleep quality during the restrictive measures following the COVID-19 lockdown (Franceschini et al., 2020). During the lockdown, the Italian National Health Service was severely struck with healthcare workers facing an overwhelming burden. In Italy, until January 5, the more recent estimate of healthcare workers deaths was 198, and 95.451 have been infected (Italian National Institute of Health, 2021). Healthcare workers are involved with infected patients’ care faced with an unknown threat to their own life. Excessive workload, fear of contagion, feeling of being under pressure, lack of specific drugs, and isolation of community were the major issues faced by healthcare workers during the time of the COVID-19 outbreak. Healthcare workers assisting patients infected with the COVID-19 may face further stress due to the stigma (Ramaci et al., 2020). On the other hand, fear of COVID-19 seems to be positively related to depression and job insecurity (Gasparro et al., 2020). However, there is still a paucity of studies investigating mental health among healthcare workers. This is surprising because the presence of mental health complaints is related to a higher reporting of insufficient workability (Ruitenburg et al., 2012). To date, many efforts by health care authorities have addressed the mental health of healthcare personnel, even though little is known on the psychological impact of the COVID-19 outbreak. Previous studies related to the 2003 outbreak of severe acute respiratory syndrome (SARS) have found a prevalence rate of severe posttraumatic stress symptoms ranging from 5 percent to 10 percent, with an increased risk for healthcare workers who had been quarantined or had worked in frontline (Bai et al., 2004; Wu et al., 2009). Although evidence on the long-term psychological impact of the COVID-19 remains unknown, early studies have provided some important results. A study involving a sample of 1257 Chinese healthcare workers reported a prevalence rate of 50.4% for depression, 44.6% for anxiety, and 71.5% for distress, with a higher risk for frontline work with COVID-19 patients in Wuhan (Lai et al., 2020). In regard to the Italian context, healthcare workers assisting patients with COVID-19 showed work-related psychological pressure, emotional exhaustion, and somatic symptoms (Barello et al., 2020). Results of a recent study highlighted the need for psychological aid interventions with anxiety and fear of contagion representing the main concern for both healthcare workers and the general population (Maldonato et al., 2020). Another study comprising a sample of 1379 Italian healthcare workers found that 49.38% expressed posttraumatic stress symptoms, 24.73% symptoms of depression, 19.80% symptoms of anxiety, and 21.90% high perceived stress (Rossi et al., 2020). High psychological distress, anxiety, and depression accounted for the need for psychological support among professionals (Conti et al., 2020). It was argued that promoting resilience should protect people from stress and psychopathological symptoms during the COVID-19 outbreak (Khan et al., 2020). Although individual characteristics could be related to mental health outcomes, no evidence is still available for healthcare workers. Bonanno (2004) described a well-consolidated theoretical and research framework that directly addressed the issue of resilience. Resilience can be defined as a stable trajectory of healthy functioning follow highly adverse and stressful events (Bonanno, 2004). In other words, resilience entails the ability to maintain a stable equilibrium while exposing to stressful and traumatic situations. Resilience is strictly related to flexibility in emotional regulation as required by the situational context (Bonanno et al., 2004). In contrast, previous theories and studies have mistakenly assumed that coping and emotion regulation strategies are always beneficial or maladaptive (Bonanno and Burton, 2013). To date, several studies have widely demonstrated that mental health depends on one’s ability to modulate emotional response under situational demands (Bonanno et al., 2004, 2018; Gupta and Bonanno, 2011; Levy-Gigi et al., 2015; Birk and Bonanno, 2016; Burton and Bonanno, 2016). Therefore, adaptation depends on one’s ability to flexibly enhance or suppress emotional expression in accordance with the contextual demands (Burton and Bonanno, 2016). The sensitivity to correct perceive contextual cues represents a key component of adaptive emotional regulatory strategies (Bonanno and Burton, 2013). Therefore, the extent to which people possess the ability to modulate emotional expression according to the context could explain how people respond to stressful events. The most of people exposed to potentially traumatic events, including the threat of an outbreak, show to be resilient and so to gain psychological adjustment (Bonanno et al., 2008). To better understand the psychological impact of the COVID-19 outbreak among healthcare workers, it is necessary to investigate resilience factors such as flexibility in emotion regulation and context sensitivity in perceive cues abilities. It could be reasonable to assume that the extent to which healthcare workers hold these characteristics could influence how they respond to stressful events such as the COVID-19 outbreak. In this perspective, a study among palliative care practitioners found that the ability to being flexible in modulating emotional response is associated with a lower risk of burnout (Lenzo et al., 2020a). Nowadays, however, no data are available about the roles of expressive flexibility and context sensitivity in the mental outcomes of healthcare workers during the COVID-19 outbreak.

The first aim of this study was to examine the prevalence of depression, anxiety, and stress among a sample of Italian healthcare workers. Consistent with other preliminary data available, we hypothesized a relevant prevalence rate for moderate to severe psychological distress. The second aim of this study was to explore the relationships between emotion regulation ability, context sensitivity, depression, anxiety, and stress. We hypothesized to find inverse relationships between emotion regulation abilities and depression, anxiety, and stress. Similarly, we expected that the ability to identify the presence and absence of stressor context cues was associated with lower levels of depression, anxiety, and stress. Finally, the third aim of this study was to investigate the role of emotion regulation ability and context sensitivity in predicting depression, anxiety, and stress. We hypothesized a significant contribution of the emotion regulation abilities and context sensitivity.

## Materials and Methods

### Participants

A sample of 218 Italian health care workers participated in this study through an online survey system without any form of compensation. Four cases were excluded for incomplete data and therefore, the final sample consisted of 214 participants. All the participants are comprised in the Sicilian Region Health Unit of the Italian National Health Service (INHS) and were recruited from April 27 to May 4, when the Italian Government has reduced restrictive measures associated with the lockdown. The inclusion criteria were being at least 18 years old and employed during the lockdown consequent to the COVID-19 outbreak with a full-time contract. *A priori* power analysis (Cohen, 1988), conducted using G^∗^Power v. 3.1.9.7 (Faul and Erdfelder, 1992), ensured the adequacy of the sample size. Hence, the sample size was computed as a function of population effect size, significance level α, statistical power, and a number of tested predictors. For these reasons, we selected the *F*-test and linear multiple regression, fixed model, and R2 increase. Therefore, we obtained a total sample size of 130 individuals (with a critical F of 2.08) by inserting a medium effect size (Cohen’s *d* = 0.15), a significant finding (at the 0.05 level), the statistical power of 0.90, and a number of 7 tested predictors.

As shown in Table 1, the final sample consisted of 130 females and 84 males working both in hospital and home care services during the COVID-19 outbreak. Participants ranged in age from 23 to 72 years (*M* = 39.58 ± 11.40). With regard to marital status, 77% was married or in a steady relationship. Twenty-five percent of the respondents were nurses (*n* = 54), 24% were physicians (*n* = 51), 16% were physiotherapists (*n* = 35), 14% were healthcare assistants (*n* = 30), 7% were clinical psychologists (*n* = 15), 5% were speech therapists (*n* = 10), 3% were social workers (*n* = 7), 5% were other health professions (*n* = 12). Also, 20% (*n* = 42) of the healthcare workers assisted COVID-19 patients.

**TABLE 1:** Demographic and work-related characteristics of the sample.

Variable	*M*	*SD*	*n*	*Percentage*
Age (in years)	39.80	11.39		
Gender				
Male			84	39.3%
Female			130	60.7%
Marital status				
Married or in a steady relationship			165	77.1%
Single, widowed, or divorced			49	22.9%
Work experience in years	11.90	10.32		
Working position				
Front-line			42	19.6%
Second-line			172	80.4%

**N* = 214.*

### Procedure

Participants were recruited through an online advertisement promoted by the Local Health Unit of the Italian National Health Service (INHS). The advertisement connected participants who were interested to an external page with information and consent to participate to this study. All participants completed the survey anonymously and gave informed consent electronically before participate. The informed consent form showed two options (yes or no). Subjects who selected “yes” accessed the survey page. Moreover, subject could leave the survey at any time. Privacy of the participants was guaranteed in accordance with the European Union General Data Protection Regulation 2016/679. The study was conducted in accordance with the 1964 Declaration of Helsinki and its later amendments. The online survey included a self-report questionnaire to collect data on age, gender, relationship status, profession role, work experience, and contact with patients with COVID-19. The study was approved by the Research Ethics Committee for Psychological Research of the University of Messina (no. 38518).

### Measures

#### Depression, Anxiety, and Stress

The Depression Anxiety Stress Scale – 21 (DASS-21) (Lovibond and Lovibond, 1995) was used to measure depression, anxiety, and stress. The DASS-21 is a 21-item self-report instrument using a four-point Likert scale ranging from “never” (0) to always (3). It consisted of three scales as follows: depression (DASS-21 Depression), assessing dysphoria, low self-esteem, anhedonia, lack of interest, and passivity (e.g., “I felt that life was meaningless”); anxiety (DASS-21 Anxiety), comprising somatic and subjective symptoms of anxiety (e.g., “I felt scared without any good reason”); stress (DASS-21 Stress), evaluating persistent arousal, irritability, psychological tension, and agitation (e.g., “I felt that I was rather touchy”). In the present study, the Italian version of DASS-21 showing excellent psychometric properties was used (Bottesi et al., 2015). Adequate levels of reliability were detected in this sample for all the three subscales (Depression, α = 0.83; Anxiety, α = 0.78; Stress, α = 0.87).

#### Emotion Regulation Ability

The Flexible Regulation of Emotional Expression (FREE) (Burton and Bonanno, 2016) scale is a 16-item self-report and scenario-based questionnaire assessing an individual’s perceived ability to modulate emotional expressions and being flexible. Regulatory flexibility is a central component for adjusting to stressful life events. The FREE Scale consisted of two relatively independent factors, which one measures the ability to enhance emotional expression (FREE Enhance ability), and the other one measures the ability to suppress emotional expression (FREE Suppress ability). Also, overall expressive flexibility (FREE Flexibility score) is calculated from the FREE Enhancement and FREE Suppression scales. All the items are rated on a 6-point Likert scale ranging from 1 (unable) to 6 (very able). Higher FREE scores are associated with greater flexibility in modulating emotional expressions.

#### Context Sensitivity

The Context Sensitivity Index (CSI) is a 20-item self-report and scenario-based questionnaire measuring context sensitivity, which is the ability to perceive cues to contextual demands across different situations (Bonanno et al., 2018). The items are rated on a 7-point Likert scale ranging from 1 (not at all) to 7 (very much). Previous studies have shown that context sensitivity is a crucial component of successful self-regulation. The CSI consists of two indices assessing the ability to capture sensitivity to the presence of contextual cues (CSI Cue Presence index) and sensitivity to the relative absence of cues (Cue Absence index). An overall CSI score (CSI Overall index) is calculated by averaging the Cue Presence and Cue Absence indices.

### Statistical Analyses

Statistical analysis was performed using IBM SPSS Statistics version 22 (IBM Corporation, Armonk, NY, United States). Data obtained from this study were checked, and descriptive and inferential statistical analyses were then carried out. Internal consistency was calculated for the DASS-21 but not for the FREE and the CSI measures because they are scenario-based indices (Bonanno et al., 2018). Indeed, each item/scenario of the FREE and the CSI measures is a unique aspect of the latent construct. An independent was used to compare the DASS-21 Depression, the DASS-21 Anxiety, and the DASS-21 Stress in the second-line healthcare workers and the front-line healthcare workers. Also, the effect size (Cohen’s *d*) was computed to quantify the difference between the second-line and front-line healthcare workers. Relationships between FREE, CSI, and DASS-21 were performed with Pearson product-moment correlation coefficients. To explore the relationship between depression, anxiety, and stress with emotion regulation ability and context sensitivity, three hierarchical regression analyses were conducted, each consisting of two steps. The DASS-21 Depression, the DASS-21 Anxiety, and the DASS-21 Stress scales were the dependent variables in all three regressions. Age, gender, and work experience were put in as covariates in both steps. In the second step, the FREE Enhance and the FREE Suppress abilities, the CSI Cue Presence and the CSI Cue Absence indices were inserted for testing if they can predict the DASS-21 Depression, the DASS-21 Anxiety, and the DASS-21 Stress scales scores among healthcare workers during the COVID-19 outbreak.

## Results

### Prevalence of Depression, Anxiety, and Stress

Table 2 shows the percentage of healthcare workers falling into each of the five categories, such as normal, mild, moderate, severe, and extremely severe based on the Lovibond and Lovibond’s percentile cut-offs (1995). The overall prevalence of moderate-to-extremely severe depression (DASS-21 Depression), anxiety (DASS-21 Anxiety), and stress (DASS-21 Stress) among participants was 8, 9.8, and 8.9%, respectively. Healthcare workers assisting patients with COVID-19 obtained scores significantly much higher than other participants on the three DASS-21 scales. We found a prevalence of moderate-to-extremely severe ranging from 21.5% for anxiety to 33.4% for stress. Moreover, Table 3 displays the result of the independent *t*-tests for the front-line healthcare workers assisting patients with COVID-19 and second-line healthcare workers. Results indicated that there were significant differences in the DASS-21 Depression [*t*(212) = 4.04, *p* < 0.001], the DASS-21 Anxiety [*t*(212) = 2.60, *p* = 0.010], and the DASS-21 Stress [*t*(212) = 4.50, *p* < 0.001]. Lastly, based on benchmarks suggested by Cohen (1988), results showed a medium effect size ranging from 0.436 for the DASS-21 Anxiety scale and 0.664 for the DASS-21 Stress scale.

**TABLE 2:** Prevalence of depression, anxiety, and stress.

DASS-21 category	DASS-21 Depression		DASS-21 Anxiety		DASS-21 Stress	
	
	Second-line HCWs	Front-line HCWs	Second-line HCWs	Front-line HCWs	Second-line HCWs	Front-line HCWs
Normal	83.6%	71.4%	85.5%	78.6%	85.5%	66.7%
Mild	8.4%	9.5%	4.7%	0%	5.6%	2.4%
Moderate	5.1%	7.1%	7.9%	19.0%	6.1%	23.8%
Severe	2.4%	11.9%	1.0%	2.4%	1.4%	2.4%
Extremely severe (98–100)	0.5%	0%	0.9%	0%	1.4%	4.8%

**N* = 214; The prevalence in each category is based on the percentiles corresponding to Lovibond and Lovibond’s cut-offs (1995).*

**TABLE 3:** Results of the *t*-tests and effect size for depression, anxiety, and stress.

Variable	Second-line HCWs (*n* = 172)		Front-line HCWs (*n* = 42)		*t*(212)	*p*	Cohen’s *d*
	*M*	*SD*	*M*	*SD*			
DASS-21 Depression	3.90	4.39	7.49	7.55	4.04	0.001	0.581
DASS-21 Anxiety	2.78	4.14	4.67	4.52	2.60	0.010	0.436
DASS-21 Stress	7.69	5.72	12.71	9.02	4.50	0.001	0.664

### Correlational Analysis

Table 4 displays descriptive statistics and correlation coefficients among the observed variables. The FREE Enhance ability was negatively associated with the DASS-21 Depression scale (*r* = −0.25; *p* < 0.01) and the DASS-21 Stress scale (*r* = −0.23; *p* < 0.01). The FREE Suppress ability was negatively associated with the DASS-21 Depression scale (*r* = −0.23; *p* < 0.01), the DASS-21 Anxiety scale (*r* = −0.15; *p* < 0.01), and the DASS-21 Stress scale (*r* = −0.27; *p* < 0.01). Also, FREE Flexibility score was negatively associated with the DASS-21 Depression scale (*r* = −0.46; *p* < 0.01), the DASS-21 Anxiety scale (*r* = −0.33; *p* < 0.01), and the DASS-21 Stress scale (*r* = −0.54; *p* < 0.01). The CSI Cue Presence index was negatively associated with the DASS-21 Depression scale (*r* = −0.14; *p* < 0.01) and the DASS-21 Anxiety scale (*r* = −0.18; *p* < 0.01). There was no significant correlation between the CSI Cue Absence index and the DASS-21 Depression, the DASS-21 Anxiety, and the DASS-21 Stress scales. Finally, the CSI Overall index was negatively associated with the DASS-21 Anxiety scale (*r* = −0.26; *p* < 0.01) but not with the DASS-21 Depression and the DASS-21 Stress scales.

**TABLE 4:** Descriptive and correlational analyses FREE, CSI, and DASS-21.

Variable	*Min*	*Max*	*M*	*SD*	1	2	3	4	5	6	7	8
1. FREE enhance ability	4	24	16.57	3.53								
2. FREE suppress ability	7	24	14.84	3.57	0.50**							
3. FREE flexibility score	16	48	30.59	6.36	0.80**	0.81**						
4. CSI Cue presence index	12	56	33.69	7.53	0.30**	0.33**	0.32**					
5. CSI cue absence index	11	60	39.18	7.95	−0.29**	−0.27**	−0.27**	−0.45**				
6. CSI overall index	24	49	36.43	4.07	−0.01	0.04	0.03	0.49**	0.56**			
7. DASS-21 depression	0	24	4.60	5.33	−0.25**	−0.23**	−0.46**	−0.14*	0.04	−0.09		
8. DASS-21 anxiety	0	26	3.15	4.27	−0.11	−0.15*	−0.33**	−0.18**	−0.09	−0.26**	0.64**	
9. DAS-21 stress	0	34	8.67	6.77	−0.23**	−0.27**	−0.54**	−0.12	0.04	−0.08	0.72**	0.67**

*N* = 214; FREE, Flexible Regulation of Emotional Expression; CSI, Context Sensitivity Index; DASS-21, Depression Anxiety Stress Scale – 21; *Min*, minimum value; *Max*, maximum value; *M*, mean; *SD*, standard deviation.*p < 0.05, **p < 0.01.

### Regression Analyses

Table 5 shows the results of the effects of FREE Enhance ability, FREE Suppress ability, CSI Cue Presence, and the CSI Cue Absence indices controlling for age, gender, and work experience on the DASS-21 Depression, the DASS-21 Anxiety, and the DASS-21 stress scales. In predicting the DASS-21 Depression scale, only age was significant at first step (β = −0.30; *p* < 0.05). In step 2, the effect of age did not persist. Also, FREE Enhance ability (β = −0.19; *p* < 0.05) was statistically significant to explain the DASS-21 Depression scores among healthcare workers with *R*^2^ reaching 0.10.

**TABLE 5:** Regression results of the effects of demographic and work-related variables, FREE, and CSI on depression, anxiety, and stress.

Predictor of DASS-21 Depression	*B*	*b* 95% CI [LL, UL]	*Beta*	*sr*^2^	*r*	Fit	Difference
(Intercept)	7.17**	[3.11, 11.23]					
Age	−0.14*	[−0.26, −0.02]	−0.30	−0.15	−0.09		
Gender	0.92	[−0.56, 2.40]	0.08	0.08	0.06		
Work experience	0.12	[−0.01, 0.25]	0.23	0.12	−0.02		
						*R*^2^ = 0.027	
(Intercept)	17.99**	[9.69, 26.28]					
Age	−0.10	[−0.22, 0.21]	−0.21	−0.11	−0.09		
Gender	0.29	[−1.18, 1.76]	0.03	0.03	0.06		
Work experience	0.08	[−0.05, 0.22]	0.16	0.08	−0.02		
FREE enhance ability	−1.16*	[−2.10, −0.21]	−0.19	−0.16	−0.25**		
FREE suppress ability	−0.67	[−1.61, 0.26]	−0.11	−0.09	−0.23**		
CSI cue presence	−0.05	[−0.15, 0.06]	−0.06	−0.05	−0.14*		
CSI cue absence	−0.05	[−0.15, 0.05]	−0.08	−0.07	0.04		
						*R*^2^ = 0.098**	Δ*R^2^* = 0.070

**Predictor of DASS-21 Anxiety**	***B***	***b* 95% CI [LL, UL]**	***beta***	***sr*^2^**	***r***	**Fit**	**Difference**

(Intercept)	5.72**	[2.49, 8.95]					
Age	−0.13**	[−0.23, −0.04]	−0.35	−0.18	−0.13*		
Gender	0.92	[−0.26, 2.09]	0.11	0.10	0.08		
Work experience	0.10	[−0.00, 0.21]	0.25	0.13	−0.05		
						*R*^2^ = 0.042*	
(Intercept)	16.76**	[10.15, 23.37]					
Age	−0.10*	[−0.20, −0.00]	−0.27	−0.13	−0.13*		
Gender	0.57	[−0.60, 1.74]	0.07	0.06	0.08		
Work experience	0.08	[−0.03, 0.18]	0.19	0.10	−0.05		
FREE enhance ability	−0.27	[−1.03, 0.48]	−0.06	−0.05	−0.11*		
FREE suppress ability	−0.41	[−1.16, 0.34]	−0.09	−0.07	−0.15**		
CSI cue presence	−0.12**	[−0.20, −0.03]	−0.21	−0.18	−0.18		
CSI cue absence	−0.13**	[−0.21, −0.05]	−0.23	−0.20	−0.09		
						*R*^2^ = 0.108**	Δ*R^2^* = 0.066

**Predictor of DASS-21 Stress**	***B***	***b* 95% CI [LL, UL]**	***beta***	***sr*^2^**	***r***	**Fit**	**Difference**

(Intercept)	13.67**	[8.66, 18.68]					
Age	−0.29**	[−0.44, −0.14]	−0.48	−0.25	−0.17**		
Gender	2.29*	[0.47, 4.11]	0.17	0.16	0.13*		
Work experience	0.23**	[0.06, 0.39]	0.35	0.18	−0.06		
						*R*^2^ = 0.082**	
(Intercept)	24.59**	[14.30, 34.87]					
Age	−0.24**	[−0.39, −0.09]	−0.41	−0.21	−0.17**		
Gender	1.63	[−0.20, 3.45]	0.12	0.11	0.13*		
Work experience	0.19**	[0.02, 0.35]	0.28	0.14	−0.06		
FREE enhance ability	−1.08	[−2.26, 0.09]	−0.14	−0.12	−0.23**		
FREE suppress ability	1.22*	[−2.38, −0.06]	−0.16	−0.13	−0.27**		
CSI cue presence	−0.01	[−0.14, 0.13]	−0.01	−0.01	−0.12*		
CSI cue absence	−0.05	[−0.17, 0.07]	−0.06	−0.05	0.04		
						*R*^2^ = 0.141**	Δ*R^2^* = 0.059

*N* = 214; FREE, Flexible Regulation of Emotional Expression; CSI, Context Sensitivity Index; DASS-21, Depression Anxiety Stress Scale – 21. A significant *b*-weight indicates the beta-weight and semi-partial correlations are also significant.*sr*^2^ represents the semi-partial correlation squared.*r* represents the zero-order correlation.*LL* and *UL* indicate the lower and upper limits of the confidence interval for B.*p < 0.05.**p < 0.01.

The second regression analyses examined the same model event though considering the DASS-21 Anxiety scale as the dependent variable. Only age (β = −0.35; *p* < 0.01) was statistically significant at step 1 and this effect persisted at step 2 (β = −0.27; *p* < 0.05). In step 2, the CSI Cue Presence index (β = −0.21; *p* < 0.01) and the CSI Cue Absence index (β = −0.23; *p* < 0.01) gave a substantial contribution in explaining the DASS-21 Anxiety scores.

Finally, the third regression analyses considered the DASS-21 Stress scale as the dependent variable. In step 1, age (β = −0.48; *p* < 0.01), gender (β = 0.17; *p* < 0.05), and work experience (β = 0.35; *p* < 0.01) were all statistically significant even though gender did not maintain this effect at step 2. In addition, FREE Suppress ability (β = −0.16; *p* < 0.05) was statistically significant with the model reaching a *R*^2^ of 0.14.

## Discussion

### Summary of the Main Findings

This study examined depression, anxiety, and stress in a sample of Italian healthcare workers facing the COVID-19 outbreak. Expressive flexibility and context sensitivity were accounted for explain depression, anxiety, and stress. During the most critical months of the COVID-19 outbreak, healthcare practitioners experienced a higher workload due to the emergency, with unknown consequences for their mental health. Although a conceivable higher impact on healthcare workers who are assisting patients with COVID-19, it is could expect a relevant psychological impact for those who are involved in the everyday assistance of patients with a chronic medical condition (Lenzo et al., 2020c; Sardella et al., 2020). First evidence indicates that a relevant percentage of healthcare workers reported mood and sleep symptoms during the COVID-19 outbreak (Pappa et al., 2020). Both medical staff and the population have experienced high levels of depression, anxiety, and stress (Lenzo et al., 2020b; Liu et al., 2020). In this perspective, the first aim of this study was to investigate the depression, anxiety, and stress levels among healthcare workers involving in the COVID-19 outbreak. Results of descriptive statistics revealed prevalence rates of moderate to extremely severe symptoms ranging from 8 percent for depression to about 10 percent for anxiety. Participants of this study had lower levels of depression, anxiety, and stress than the prevalence reported by early studies involving healthcare workers (Conti et al., 2020; Lai et al., 2020; Rossi et al., 2020). These findings could have depended on assisting patients with COVID-19 leading to an increased fear of being infected. A further exploratory analysis was performed to examine depression, anxiety, and stress among healthcare workers who assisted patients infected. We detected higher prevalence rates of moderate-to-extremely severe when considering healthcare workers assisting patients with COVID-19, even though we assumed these results as explored. Although encouraging due to the paucity of studies on this topic, future research involving a well-balanced sample should examine in deep the results we obtained. In contrast, other studies have found lower prevalence rates. One study involving a large sample of healthcare workers reported prevalence rates of moderate to very severe symptoms of 5 percent for depression, 9 percent for anxiety, and 2 percent for stress (Chew et al., 2020). Resilience factors could be useful to understand the difference in prevalence rates among these largely cross-sectional studies investigating depression, anxiety, and stress among healthcare workers during the COVID-19 outbreak (Chen and Bonanno, 2020). Since the efficacy of coping strategies varies across the different contexts, it is worthwhile to point out the importance of flexibility (Bonanno et al., 2004; Bonanno and Burton, 2013). Indeed, it was demonstrated that mental health depends on one’s ability to flexibly enhance or suppress emotional response under situational demands (Burton and Bonanno, 2016). A prerequisite for efficacious self-regulation and adaption consists in the ability to correctly perceive cues to contextual demands across different situations (Bonanno et al., 2018). We examined these key components of resilience using the FREE Scale and the CSI in a sample of Italian healthcare workers facing the COVID-19 outbreak.

In this context, the second aim of this study was to examine the relationship between emotion regulation ability, context sensitivity, depression, anxiety, and stress. Previous research involving healthy subjects found inverse relationships between emotion regulation ability, depression, and anxiety (Burton and Bonanno, 2016; Chen et al., 2018). Our study’s findings confirmed these relationships and added evidence to the fallacy of uniform efficacy when considering the efficacy of coping and emotion regulation strategies (Bonanno and Burton, 2013). However, regulatory flexibility is subsequent to the ability to perceive or not perceive contextual cues named as “context sensitivity” (Bonanno, 2004). Specifically, the ability to perceive contextual cues when appropriate was found to be associated with emotion regulation and flexibility in coping response (Bonanno et al., 2018). Consistent with these findings, we found positive associations between cue presence ability and flexibility in emotional response among healthcare workers. We also found that cue presence was inversely associated with depression and anxiety highlighting its role in psychopathology. Conversely, cue absence ability was related to cue presence but not with flexibility. It is worthwhile to point out that the ability to decide when a contextual cue is not present is less clearly associated with the cue presence ability and flexibility in emotional response (Bonanno et al., 2018).

The third aim of this study was to explore the role of enhancement and suppression abilities, and cue presence and cue absence abilities on depression, anxiety, and stress. Although the considerable amount of evidence on flexibility and context sensitivity, there is still a paucity of studies taking into account these factors among healthcare workers. This is surprising because understanding the role of flexibility and context sensitivity can help to explain the prevalence rates of depression, anxiety, and stress, and so implement interventions to prevent them. Recently, a study found that the ability to flexibility enhance or suppress emotional response decreases burnout risk in the context of palliative home care (Lenzo et al., 2020a). We hypothesized that these abilities would significantly influence depression, anxiety, and stress among a sample of healthcare workers are facing the COVID-19 outbreak. Our study’s findings revealed that being flexible in emotional response and context sensitivity are differently associated with depression, anxiety, and stress. It is worthwhile to highlight that demographic and work-related factors had a relevant role only in predicting stress among healthcare workers. More specifically, our results showed a significant effect of the perceived individual’s ability to enhance emotional expression on depression. Lower levels of enhancement ability are related to social functioning deficits but not to depression among health subjects (Burton and Bonanno, 2016). This result could have partially depended on the sample characteristics. Another study involving a small sample of combat veterans pointed out that enhancement ability, but not suppression ability, was associated with greater symptoms of depression and posttraumatic stress disorder (Rodin et al., 2017). Nonetheless, we found different results when considering anxiety. In predicting anxiety, we found a significant role in the ability named context sensitivity (Bonanno and Burton, 2013). The ability to correctly perceive contextual cues represents a prerequisite for efficacious self-regulation and adaptation. Findings from a recent study reported that cue presence and cue absence were associated with anxiety (Bonanno et al., 2018). Interestingly, the authors argued that psychopathology may concern the failure to read key contextual cues in a specific situation, as well as what to do not consider in other situations. An analogous point of view can be adopted to consider anxiety among healthcare workers, even though more research is needed in this context. Finally, our findings pointed out a significant role for suppression ability in predicting stress. This finding adds evidence for the different role enhancement and suppression abilities for psychological health. While enhancement ability allows emotional signals, which may favor better interpersonal relationships, suppression ability may be central for decrease psychological distress since a deficit in response inhibition has been involved as a risk factor for a wide array of psychopathology, comprising anxiety and depression (Warren et al., 2013). Although the three regression models revealed a significant role for flexibility and context sensitivity in predicting reported symptoms of depression, anxiety, and stress, a considerable number of aspects have been demonstrated to enhance resilience, such as personality factors and social support (Bonanno, 2004). Future research including these variables in the regression analyses could explain a higher percentage of variance for psychological distress among healthcare workers. Nonetheless, taken together these findings emphasized the interplay of expressive flexibility and context sensitivity with depression, anxiety, and stress. Consequently, relevant clinical implications of this study concern the possibility to implement prevention interventions decreasing the psychological impact of working in adverse conditions as during the COVID-19 outbreak. In this vein, our results have shown higher depression, anxiety, and stress levels in front-line healthcare workers than in second-line healthcare workers. Findings of this study could also help to implement psychological interventions for healthcare workers assisting patients with the COVID-19 and to mitigate its psychological consequences.

### Limitations

This study has some limitations that should be addressed by future research and considered in understanding the results. First, this study adopted a cross-sectional design that did not allow us to determine causal relationships between the investigated variables. Longitudinal studies would better clarify the long-lasting impact of resilience components on depression, anxiety, and stress development among healthcare workers who faced the COVID-19 outbreak. The authors are currently carrying out this kind of research. Second, this study involved convenience sample recruitment that could have limited the generalizability of the results. The oversampling of some characteristics (i.e., gender or occupation) may not be representative of the Italian healthcare workers population. Thus, some characteristics among the respondents (i.e., profession type) could influence the results obtained. In fact, collecting data through an online survey did not permit to assess for preexisting psychiatric disorders. Nonetheless, our choice was the only solution to collect data during the Italian lockdown. The third limitation regards the use of self-assessment measures of depression, anxiety, and stress. Although the DASS-21 is a reliable and widely used instrument, social desirability could affect results. Conversely, the FREE scale and CSI are scenario-based indices that did not presuppose respondents to possess an exact awareness of their own abilities. It should be noted that both the use of self-report measures and the collection of data through an on-line survey gave us information on depression, anxiety, and stress symptoms, as reported by the participants to this study. However, in no case it is possible to state of psychiatric diagnoses that require other sources of data, as the clinical judgment.

## Conclusion

Healthcare workers were deeply involved in contrasting the COVID-19 during the Italian lockdown. Although the psychological impact of restrictive measures among the population is well documented, there is still a lack of studies focused on the consequences for healthcare workers. Our study’s results highlighted that about ten percent of participants reported moderate to extremely severe symptoms of depression, anxiety, and stress during the COVID-19 lockdown. Flexibility in emotional response and the ability to correctly perceive or not perceive contextual cues seem to explain differences in the experienced severity of these symptoms. Given these results, prevention intervention based on these resilience components could help reduce depression, anxiety, and stress among healthcare workers are facing the COVID-19 outbreak. However, there are some limitations such as the cross-sectional design that should be addressed by future research to clarify the long-term effects of flexibility and context sensitivity.

## Data Availability Statement

The raw data supporting the conclusions of this article will be made available by the authors, without undue reservation.

## Ethics Statement

The studies involving human participants were reviewed and approved by the Research Ethics Committee for Psychological Research of the University of Messina (no. 38518). The patients/participants provided their written informed consent to participate in this study.

## Author Contributions

VL and MQ provided contributions to the conception of the work, deep analysis of the literature, study design, and final approval of the manuscript. VL contributed to data analysis and writing the first draft. AS and GM contributed to the revision of the work and agreement for final approval of the manuscript. MQ and GB contributed to supervision and approved the final version of the manuscript. All authors contributed to the article and approved the submitted version.

## Conflict of Interest

The authors declare that the research was conducted in the absence of any commercial or financial relationships that could be construed as a potential conflict of interest.
